# Entinostat reverses P-glycoprotein activation in snail-overexpressing adenocarcinoma HCC827 cells

**DOI:** 10.1371/journal.pone.0200015

**Published:** 2018-07-06

**Authors:** Takumi Tomono, Tatsuya Machida, Hiroki Kamioka, Yumi Shibasaki, Kentaro Yano, Takuo Ogihara

**Affiliations:** 1 Laboratory of Clinical Pharmacokinetics, Graduate School of Pharmaceutical Sciences, Takasaki University of Health and Welfare, Takasaki-shi, Gunma, Japan; 2 Laboratory of Biopharmaceutics, Faculty of Pharmacy, Takasaki University of Health and Welfare, Takasaki-shi, Gunma, Japan; University of South Alabama Mitchell Cancer Institute, UNITED STATES

## Abstract

Epithelial-to-mesenchymal transition (EMT) in cancer cells facilitates tumor progression by promoting invasion and metastasis. Snail is a transcriptional factor that induces EMT, while P-glycoprotein (P-gp) is an efflux transporter involved in anticancer drug resistance, and P-gp efflux activity is stimulated in Snail-overexpressing lung cancer cells with EMT characteristics. Since the histone deacetylase (HDAC) inhibitor entinostat (Ent) reverses EMT features, our aim in this study was to determine whether Ent also suppresses P-gp activation in Snail-induced cells. First, we confirmed that Ent treatment reduced migration activity, downregulated E-cadherin and upregulated vimentin at the mRNA level in Snail-overexpressing cells, thus inhibiting EMT. Efflux and uptake assays using rhodamine123 (Rho123), a fluorescent P-gp substrate, showed that Ent also inhibited Snail-induced activation of P-gp. Moreover, P-gp activity was more strongly inhibited by Ent in Snail-overexpressing cells than in Mock cells. When we evaluated the uptakes of Rho123 by LLC-PK1 cells and P-gp-overexpressing LLC-GA5COL150 cells, Rho123 accumulation in LLC-GA5COL150 cells was significantly decreased compared with that in LLC-PK1 cells. Coincubation with Ent had no effect on Rho123 accumulation in either of the cell lines. Thus, Ent appears to be an inhibitor, but not a substrate, of P-gp at low concentration. Our results suggest that Ent treatment might suppress not only Snail-induced cancer malignant alteration, but also P-gp-mediated multidrug resistance.

## Introduction

Lung cancer is a leading cause of cancer death worldwide [1], in part because of its high metastatic potential [2], which is related to the occurrence of epithelial-to-mesenchymal transition (EMT) of cancer cells [3] [4]. Conversion of epithelial cancer cells to mesenchymal cancer cells (i.e., EMT) involves down-regulation of epithelial markers such as E-cadherin [5], occludin [6] and claudin [7] and up-regulation of mesenchymal markers such as vimentin [8] and ZEB1 [9]. Snail is a transcriptional factor that regulates cancer EMT through the inhibition of E-cadherin expression [10]. It is also reported that EMT triggers cancer multidrug resistance (MDR) by causing changes in the activities of drug-metabolizing enzymes [11] and drug transporters [12]. Among these transporters, P-glycoprotein (P-gp) mediates efflux of drugs, toxic compounds, xenobiotics and metabolites, and plays a key role in cancer MDR [13]. We have shown that the efflux activity, though not the expression level, of P-gp is enhanced in Snail-overexpressing lung adenocarcinoma HCC827 cells [12]. Therefore, we considered that inhibitors of cancer EMT might also be useful for overcoming cancer MDR mediated by P-gp. Entinostat (Ent) is a histone deacetylase (HDAC) inhibitor that is under clinical trial for the treatment of various cancers, such as breast cancer [14], non-small cell lung cancer [15], melanoma [16] and various solid tumors [17]. Shah et al. have revealed that Ent reverses cancer EMT characteristics *in vitro*. Moreover, they also indicated that the mechanism of EMT inhibition by Ent partially depended on Snail down-regulation [18]. Therefore, we considered that Ent might be a useful drug not only for inhibition of EMT, but also for inhibition of P-gp activation in Snail-overexpressing cells. In this context, the aim of the present study was to establish whether or not Ent inhibits P-gp activation, as well as EMT, in Snail-overexpressing cells.

## Materials and methods

### Materials and cells

Ent was purchased from MedChem Express (QLD, Australia), Rhodamine123 (Rho123) from Sigma-Aldrich (St. Louis, MO), and elacridar (Elc) from Santa Cruz Biotechnology (Santa Cruz, CA). All other reagents were commercial products of reagent grade.

Human non-small cell lung cancer cell line HCC827 was purchased from American Type Culture Collection (Manassas, VA). Porcine kidney epithelial cell line LLC-PK1 and its P-gp-overexpressing variant LLC-GA5COL150 (MDR1 gene-transfected cells) were purchased from RIKEN Cell Bank (Ibaraki, Japan). The cells were cultured in Dulbecco’s modified Eagle’s medium containing 10% fetal bovine serum (FBS), 100 units/mL penicillin, 0.1 mg/mL streptomycin. LLC-GA5COL150 cells were cultured in the same medium supplemented with 500 μg/mL geneticin and 150 ng/mL colchicine. All cells were cultured in a humidified atmosphere of 5% CO_2_ at 37°C.

### Adenoviral vector infection and Ent treatment of HCC827 cells

Mock or Snail human adenovirus serotype 5 vectors (Ad) were gifts from Masahiro Kajita [19]. Purification and particle counting of Ad were carried out as described [12].

HCC827 cells were seeded at 0.5 × 10^5^ on 24-well culture plates. One day after seeding, cells were infected with Ad at 1000 VPs/cell. After 3 days, the medium was changed to fresh medium with or without 10 nM Ent. After 4 days, cells were used for RNA extraction, Rho123 efflux assay and uptake assay.

### Quantitative real-time polymerase chain reaction

Total RNA was isolated from HCC827 cells using TRIzol^TM^ reagent (Invitrogen, Carlsbad, CA) and 1 μg of extracted total RNA was used for cDNA synthesis with ReverTra Ace^®^ (Toyobo, Osaka, Japan).

Quantitative real-time polymerase chain reaction (qRT-PCR) was carried out using Power SYBR^TM^ Green PCR Master Mix (Applied Biosystems, Carlsbad, CA) with an Mx3000P qPCR system (Agilent Technologies, Santa Clara, CA). The values of fold change in mRNA expression were calculated by the 2-ΔΔ threshold cycle (Ct) method, normalized to glyceraldehyde 3-phosphate dehydrogenase (GAPDH). Oligonucleotide primers for E-cadherin, vimentin, Snail, P-gp and GAPDH were as follows: E-cadherin (159 bp), F: 5’-CAGCACGTACACAGCCCTAA-3’, R: 5’-ACCTGAGGCTTTGGATTCCT-3’, vimentin (105 bp), F: 5’-CGGGAGAAATTGCAGGAGGA-3’, R: 5’-AAGGTCAAGACGTGCCAGAG-3’, Snail (249 bp), F: 5’- GAAAGGCCTTCAACTGCAAA-3’, R: 5’-TGACATCTGAGTGGGTCTGG-3’, P-gp (157 bp), F: 5’- CCCATCATTGCAATAGCAGG-3’, R: 5’-GTTCAAACTTCTGCTCCTGA-3’, GAPDH (87 bp), F: 5’-TGCACCACCAACTGCTTAGC-3’, R: 5’-GGCATGGACTGTGGTCATGAG-3’.

### Cell viability assay

One day after HCC827 cell seeding at 1 × 10^4^ cells/well on a 96-well culture plate, the medium was replaced with medium containing 0–100 μM Ent. After 4 days, alamarBlue^®^ reagent (AbD Serotec, Dusseldorf, Germany) was added and the absorbance was measured at 570 and 600 nm. The viability was calculated from these values in accordance with the manufacturer’s instructions.

### Western blot analysis

One day after HCC827 cell seeding at 2.5 × 10^5^ cells/well on a 6-well culture plate, the medium was replaced with medium containing 0–1000 nM Ent. After 4 days, the cells were washed twice with ice-cold phosphate-buffered saline (PBS), collected by trypsinization, washed twice with ice-cold PBS, and lysed with RIPA Buffer (Wako, Osaka, Japan) containing cOmplete Mini, EDTA-free (Roche, Basel, Switzerland) for 30 min on ice. The lysate was centrifuged at 15,000*g* for 30 min at 4°C and the supernatant was collected. Subsequent processing was carried out as described elsewhere [12]. Briefly, a 30 μg aliquot of protein was separated on 4–20% polyacrylamide gel and transferred onto a polyvinylidene difluoride membrane. The membrane was blocked with 5% skim milk and blotted with primary antibodies [anti-histone H3 (BioLegend, San Diego, CA) or anti-acetyl-histone H3 (Lys9) (Cell Signaling Technology, Danvers, MA)] overnight at 4°C. The next day, the membrane was washed and reacted with goat anti-rabbit IgG-HRP (Santa Cruz Biotechnology, Santa Cruz, CA) for 1 hr at room temperature. The band intensity was measured using ECL substrate (GE Healthcare, Little Chalfont, Buckinghamshire).

### Migration assay

Migration assay was carried out according to the Boyden chamber method in Transwells with polycarbonate membranes having 8-μm pore size (Corning, MA, USA). Briefly, cells were seeded at 1 × 10^6^ cells/dish on 10 cm cell culture dishes. The next day, cells were infected with Ad at 1000 VPs/cell. After 3 days, cells were suspended in FBS-free medium and seeded at 5 × 10^4^ cells/chamber on the Transwell in the presence or absence of 10 nM Ent. Medium including 10% FBS with or without 10 nM Ent was added under the chamber. After 4 days, cells were washed twice with ice-cold phosphate-buffered saline (PBS) and fixed with 3.7% formaldehyde for 2 min at room temperature. Cells were washed twice with ice-cold PBS and permeabilized with methanol for 20 min at room temperature. Cells were stained with crystal violet solution for 15 min at room temperature. Cells on the chamber were removed with a cotton swab to identify migrated cells, which were observed by microscopy.

### Rho123 efflux assay and uptake assay

Rho123 efflux assay was carried out as described [12], with some modifications. After Ad infection and Ent treatment, HCC827 cells were washed twice with ice-cold PBS. Ice-cold 10 μM Rho123 in Opti-MEM^®^ was then added. The cells were incubated for 15 min at 4°C, then washed twice with ice-cold PBS, and further incubated in Opti-MEM^®^ for 30 min at 37°C. The cells were washed three times with ice-cold PBS and dried. Initial uptake of Rho123 in each group was evaluated using cells incubated with Rho123 for 15 min at 4°C. The efflux rate was calculated according to the following formula.

$$\begin{array}{l} \mathrm{Efflux}\;\mathrm{rate}\;\left(\mathrm{nmol}/\min/g\;\mathrm{protein}\right)=\{\mathrm{Initial}\;\mathrm{uptake}\;\mathrm{of}\;\mathrm{Rho}123\;\left(\mathrm{nmol}/g\;\mathrm{protein}\right)\;–\\ \mathrm{Residual}\;\mathrm{amount}\;\mathrm{of}\;\mathrm{Rho}123\;\mathrm{after}\;30\;\min\;\mathrm{incubation}\;\left(\mathrm{nmol}/g\;\mathrm{protein}\right)\}\;/30\;\left(\min\right) \end{array}$$

Rho123 uptake assay was carried out as described [12]. After Ad infection and Ent treatment, HCC827 cells were washed twice with Hank's balanced salt solution (HBSS) buffered with 4-(2-hydroxyethyl)-1-piperazine-ethanesulfonic acid (HEPES) (pH 7.4) (HBSS-HEPES) at 37 ^o^C. Then, HBSS-HEPES including 10 μM Rho123 was added. The cells were incubated for 1 hr at 37°C, then washed three times with ice-cold PBS, and dried.

### Rho123 uptake assay with LLC-PK1 and LLC-PK1COL150 cells

LLC-PK1 or LLC-PK1COL150 cells were seeded (5 × 10^4^ cells/well) on 24-well culture plates. After 4 days, the medium was changed to fresh medium without geneticin and colchicine. The next day, uptake experiments were carried out using 10 μM Rho123 with or without 10 nM Ent or 10 μM Elc as a P-gp inhibitor. The uptake experiments were performed as described for Rho123 uptake assay. Cells were incubated for 30 min at 37°C.

### Determination of intracellular concentrations of Rho123 and Ent

Intracellular Rho123 concentration in efflux or uptake assays was determined according to the following protocol. After experiments, cells were lysed with 0.1 N NaOH and the lysate was transferred to a 96-well black microplate. Rho123 fluorescence was measured with an ARVO^TM^MX (PerkinElmer, Waltham, MA) at wavelengths of 485 nm (excitation) and 538 nm (emission). Protein concentration in each lysate was evaluated with the DC^TM^ Protein Assay (BIO-RAD, Hercules, CA).

### Evaluation of ATPase activity in P-gp-expressing vesicles

The ATPase activity of P-gp was determined using a PREDEASY^TM^ ATPase assay kit in accordance with manufacturer’s protocol and a previous report [20]. Briefly, solutions of various concentrations of Ent (0.001–100 μM) in 2% DMSO were used for activation and inhibition assays. A suspension of P-gp-expressing membrane vesicles derived from *Spodoptera frugiperda* ovarian cells (Sf9) was incubated with the Ent solution in the presence of 2 mM MgATP for 10 min at 37°C. Vanadate was used in the activation study to evaluate background activity and verapamil was used in the inhibition study as an ATPase activator for P-gp-mediated transport. The assays were stopped by addition of the developer solution. Blocker solution was then added, and the samples were incubated for 30 min. The optical density (OD) was measured at 590 nm with a microplate reader Sunrise^TM^ rainbow RC-R (TECAN, Kanagawa, Japan). Percentage ATPase activity was calculated from the OD values.

### Statistical analysis

All data are presented as mean ± standard deviation (S.D.). Statistical analysis was undertaken using the Williams test or Holm test.

## Results

### Selection of a suitable concentration of Ent for studies of its effect on P-gp activation in HCC827 cells

**Fig 1:**
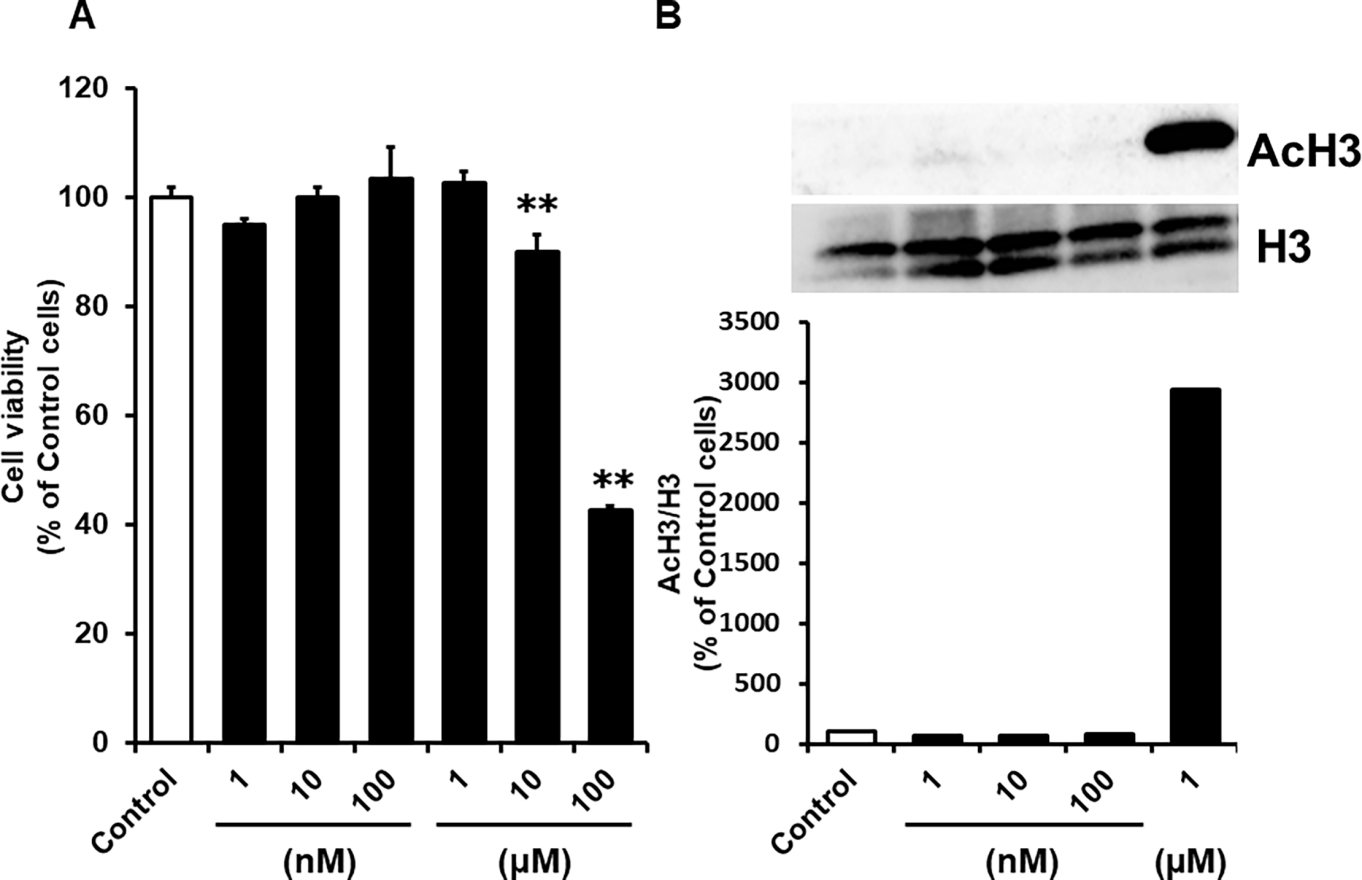
Effects of Ent on cell viability (A) and AcH3 expression (B) in HCC827 cells. (A) HCC827 cells were treated with Ent for 4 days in vitro. Cell viability was determined using alamarBlue^®^ reagent. Each value is the mean ± S.D. (n = 5). Statistical significance was evaluated with the Williams test: **p<0.01. (B) Western blot analysis of AcH3 and H3 in HCC827 whole cell lysate. Cells were treated with Ent for 4 days in vitro. Band densities were determined with a Luminescent Image Analyzer LAS-3000, and AcH3 densities were normalized by H3.

We assessed the maximum non-cytotoxic concentration and the maximum non-effective concentration of Ent using alamarBlue^®^ assays and western blotting of acetylated histone H3 (AcH3), respectively, in order to find a suitable concentration for reversing Snail-induced EMT in HCC827 cells without the need to consider either P-gp activity changes owing to Ent-induced cytotoxicity or altered AcH3 status. AlamarBlue^®^ assays revealed that 10 and 100 μM Ent exposure significantly reduced HCC827 viability (89.9 ± 3.6% in 10 μM and 42.7 ± 0.9% in 100 μM respectively). At concentrations under 1 μM, Ent did not show cytotoxicity (Fig 1A). Moreover, whereas 1000 nM Ent treatment increased AcH3 expression, 100 nM Ent had no effect on AcH3 compared with the control (Fig 1B). Therefore in subsequent studies, we used 10 nM Ent to reverse Snail-induced EMT in HCC827 cells.

### Effect of Ent on migration activity of HCC827 cells

**Fig 2:**
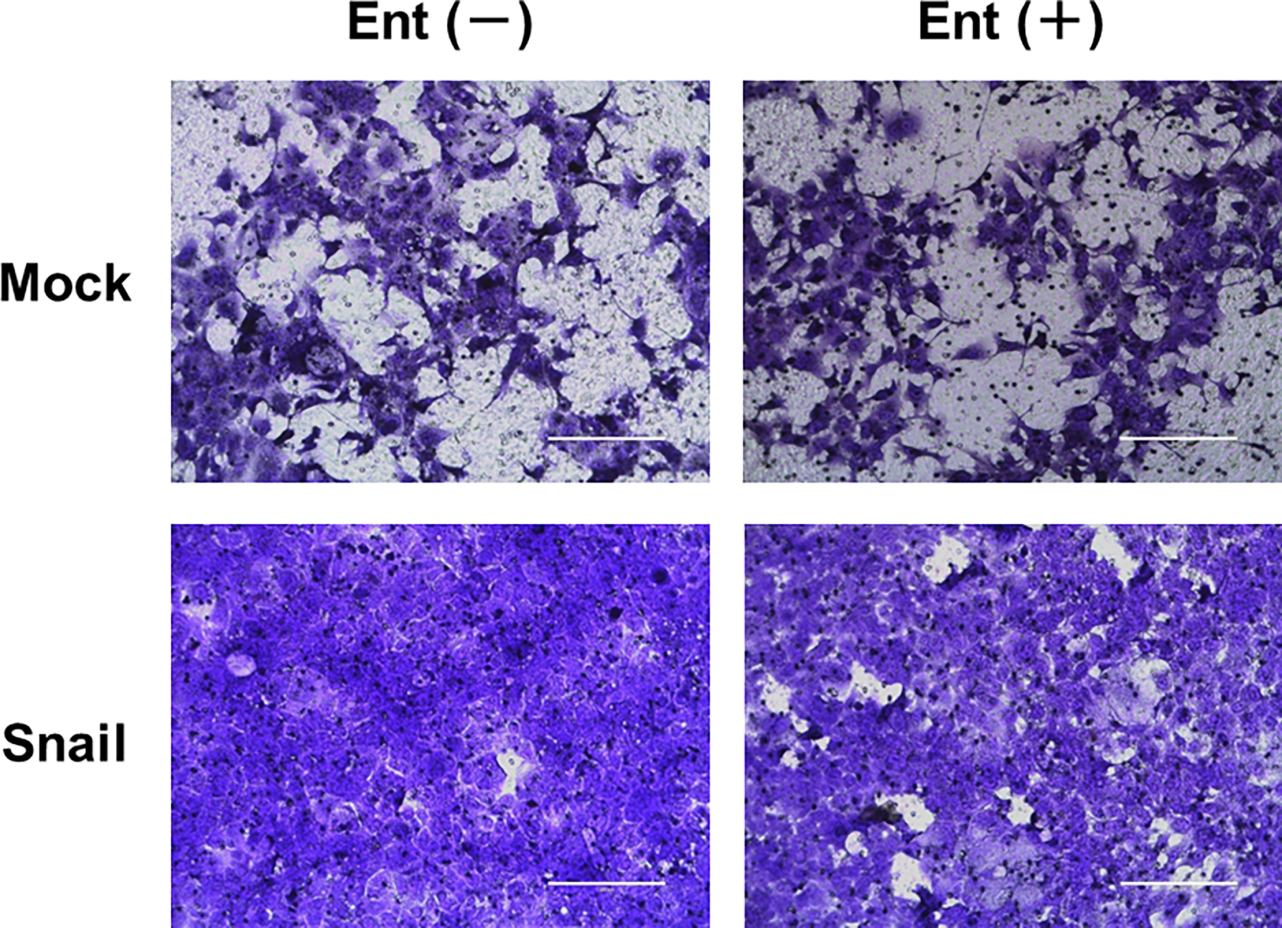
Effects of Ent on migration activity of HCC827 cells. Migration activity of Mock and Snail-overexpressing cells was assessed at day 4 with or without Ent treatment by means of Transwell^Ⓡ^ assay. Cells were stained with crystal violet. Scale bars indicate 200 μm.

Firstly, we evaluated whether Ent treatment inhibited migration activity of HCC827 cells by means of migration assay. Snail-overexpressing cells showed high migration activity. The Ent-treated Snail-overexpressing cells tended to show lower migration activity than Snail-overexpressing cells without Ent treatment, whereas the migration activity of Mock cells was not affected by Ent (Fig 2).

### Reversing effect of Ent on Snail-induced EMT in HCC827 cells

**Fig 3:**
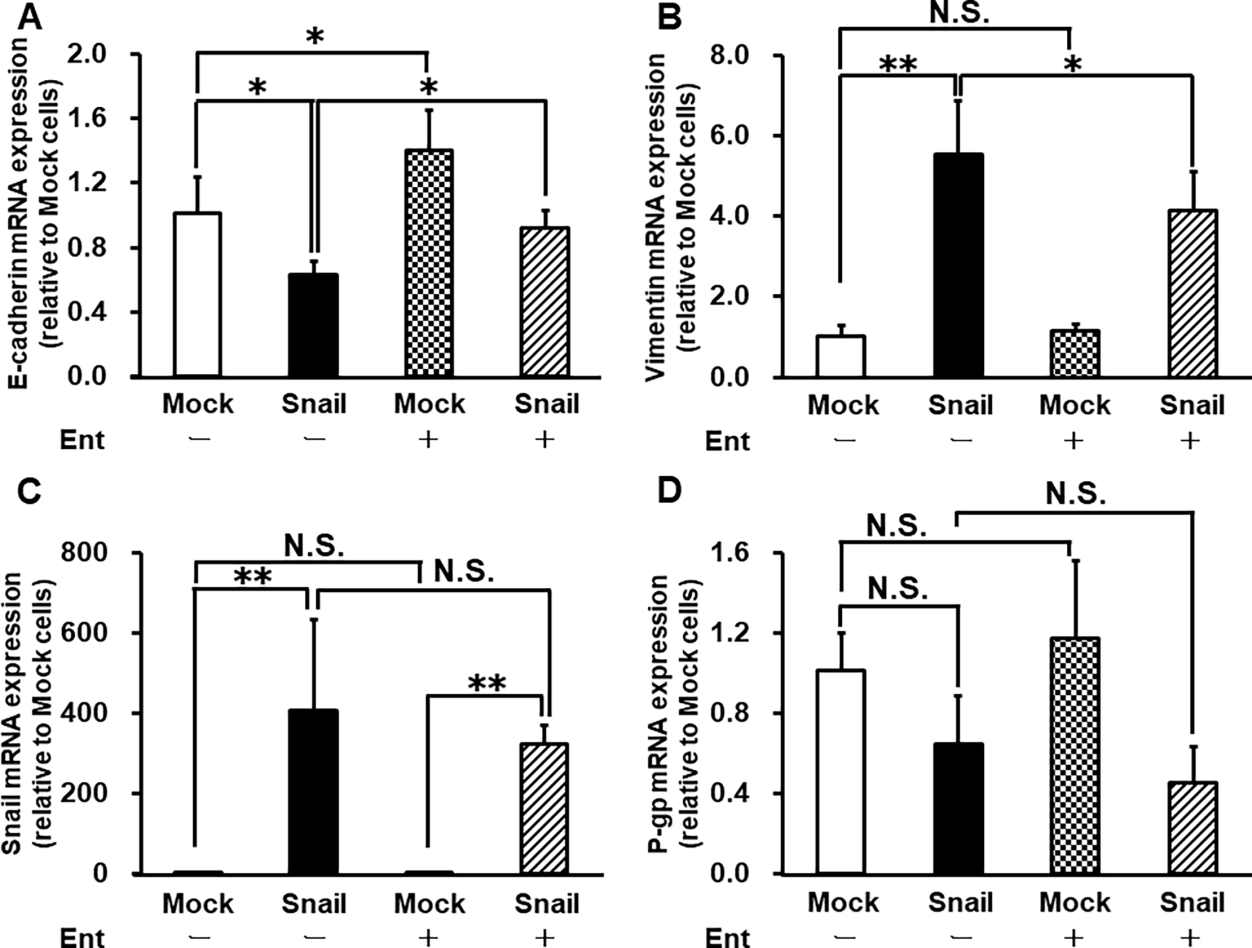
Effects of Ent on E-cadherin (A), vimentin (B), Snail (C) and P-gp (D) mRNA expression levels in HCC827 cells. Cells were infected with Ad vector for 3 days and then treated with 10 nM Ent-containing medium for 4 days in vitro. Each value is the mean ± S.D. (n = 4 for E-cadherin, n = 5 or 6 for vimentin, Snail and P-gp). Statistical significance was evaluated with the Holm test: *p<0.05, **p<0.01. N.S. indicates no significant difference.

Next, we confirmed that Ent reverses Snail-induced EMT in HCC827 cells. In Snail-overexpressing cells, E-cadherin expression was reduced and vimentin expression was increased compared with Mock cells (0.63 ± 0.09 and 5.54 ± 1.34 relative to Mock cells, respectively), in agreement with our previous findings [12]. Ent treatment restored E-cadherin expression and reduced vimentin expression in Snail-overexpressing cells (0.92 ± 0.11 and 4.13 ± 0.98 relative to Mock cells, respectively) (Fig 3A and 3B). On the other hand, Snail mRNA expression was unaffected by Ent (1.03 ± 0.27 in Mock cells, 406.57 ± 225.05 in Snail-overexpressing cells, 0.76 ± 0.15 in Mock cells with Ent, and 322.06 ± 47.64 in Snail-overexpressing cells with Ent) (Fig 3C). Moreover, Snail-overexpressing cells showed lower P-gp mRNA expression than Mock cells. However, Ent treatment did not affect P-gp expression in either Mock or Snail-overexpressing cells (1.01 ± 0.18 in Mock cells, 0.65 ± 0.24 in Snail-overexpressing cells, 1.17 ± 0.39 in Mock cells with Ent, and 0.46 ± 0.18 in Snail-overexpressing cells with Ent) (Fig 3D).

### Ent suppresses P-gp activation in Snail-overexpressing HCC827 cells

**Fig 4:**
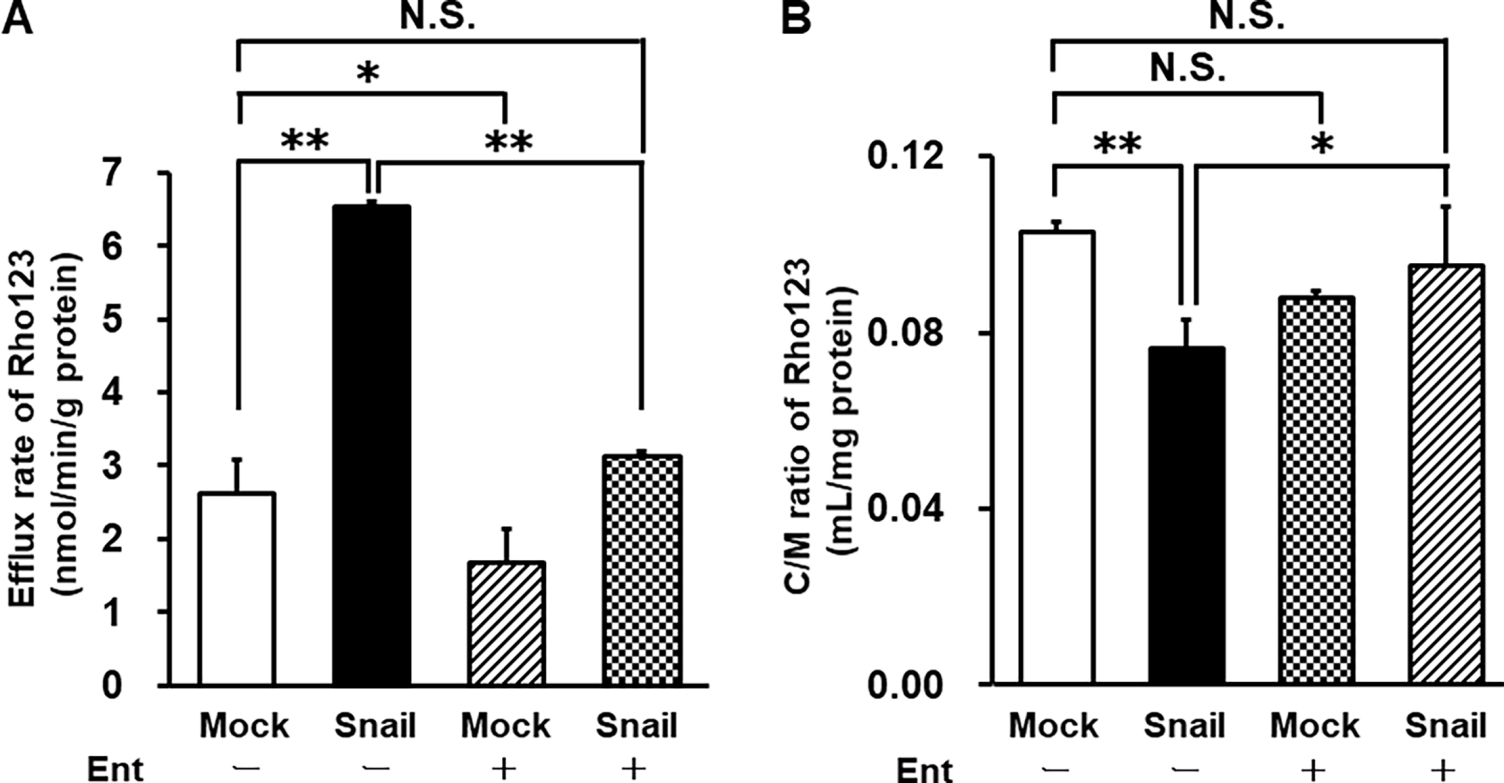
Effects of Ent on Rho123 uptake and efflux in HCC827 cells. P-gp transport activity was examined by means of efflux (A) and uptake (B) assays. In efflux assay, 10 μM Rho123 was loaded into HCC827 cells for 15 min at 4°C and the cells were then incubated in Opti-MEM^®^ without Rho123 for 30 min at 37°C. In uptake assay, HCC827 cells were incubated with 10 μM Rho123 for 60 min at 37°C. Each value is the mean ± S.D. (n = 3–5 for uptake assay and n = 3 for efflux assay). Statistical significance was evaluated with the Holm test: *p<0.05, **p<0.01. N.S. indicates no significant difference.

We previously showed that P-gp is activated in EMT-induced HCC827 cells [12]. Therefore, we evaluated the ability of Ent to block P-gp activation. Firstly, we carried out Rho123 efflux experiments. The Rho123 efflux rate was increased in Snail-overexpressing cells. On the other hand, P-gp activity was decreased by Ent treatment in both Mock and Snail-overexpressing cells (2.61 ± 0.48 nmol/min/g protein in Mock cells, 6.54 ± 0.08 nmol/min/g protein in Snail-overexpressing cells, 1.66 ± 0.48 nmol/min/g protein in Mock cells with Ent, and 3.13 ± 0.08 nmol/min/g protein in Snail-overexpressing cells with Ent) (Fig 4A). Rho123 uptake experiments revealed that the accumulation of Rho123 in Snail-overexpressing cells was decreased compared with that in Mock cells (0.103 ± 0.002 mL/mg protein in Mock cells and 0.077 ± 0.006 mL/mg protein in Snail-overexpressing cells). The decrease of Rho123 accumulation in Snail-overexpressing cells was reversed by Ent treatment (0.095 ± 0.014 mL/mg protein). However, Ent treatment had no effect on Rho123 accumulation in Mock cells (0.088 ± 0.001 mL/mg protein) (Fig 4B).

### Evaluation of the influence of Ent on P-gp efflux activity

**Fig 5:**
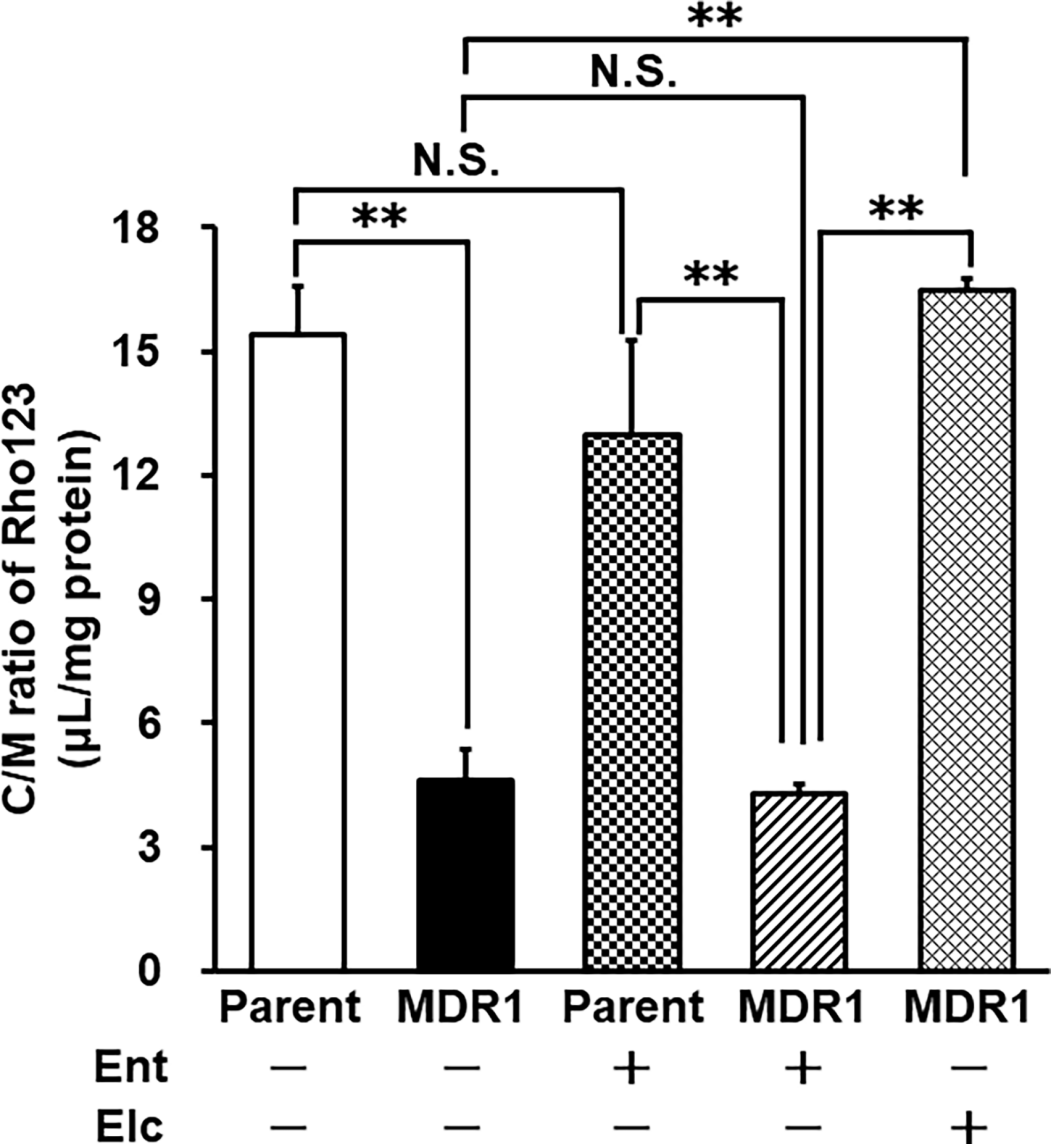
Effect of Ent on Rho123 accumulation in LLC-PK1 and LLC-GA5COL150 cells. LLC-PK1 and LLC-GA5COL150 cells were incubated with 10 μM Rho123 with or without 10 nM Ent or 10 μM Elc for 30 min at 37°C. Each value is the mean ± S.D. (n = 6). Statistical significance was evaluated with the Holm test: **p<0.01.

Finally, we examined whether 10 nM Ent inhibits Rho123 transport by P-gp. The accumulation of Rho123 in LLC-GA5COL150 cells was significantly decreased compared with that in LLC-PK1 cells. Coincubation with Ent had no effect on Rho123 accumulation in either of the cell lines. However, the P-gp inhibitor Elc significantly increased Rho123 uptake in LLC-GA5COL150 cells (Fig 5).

**Fig 6:**
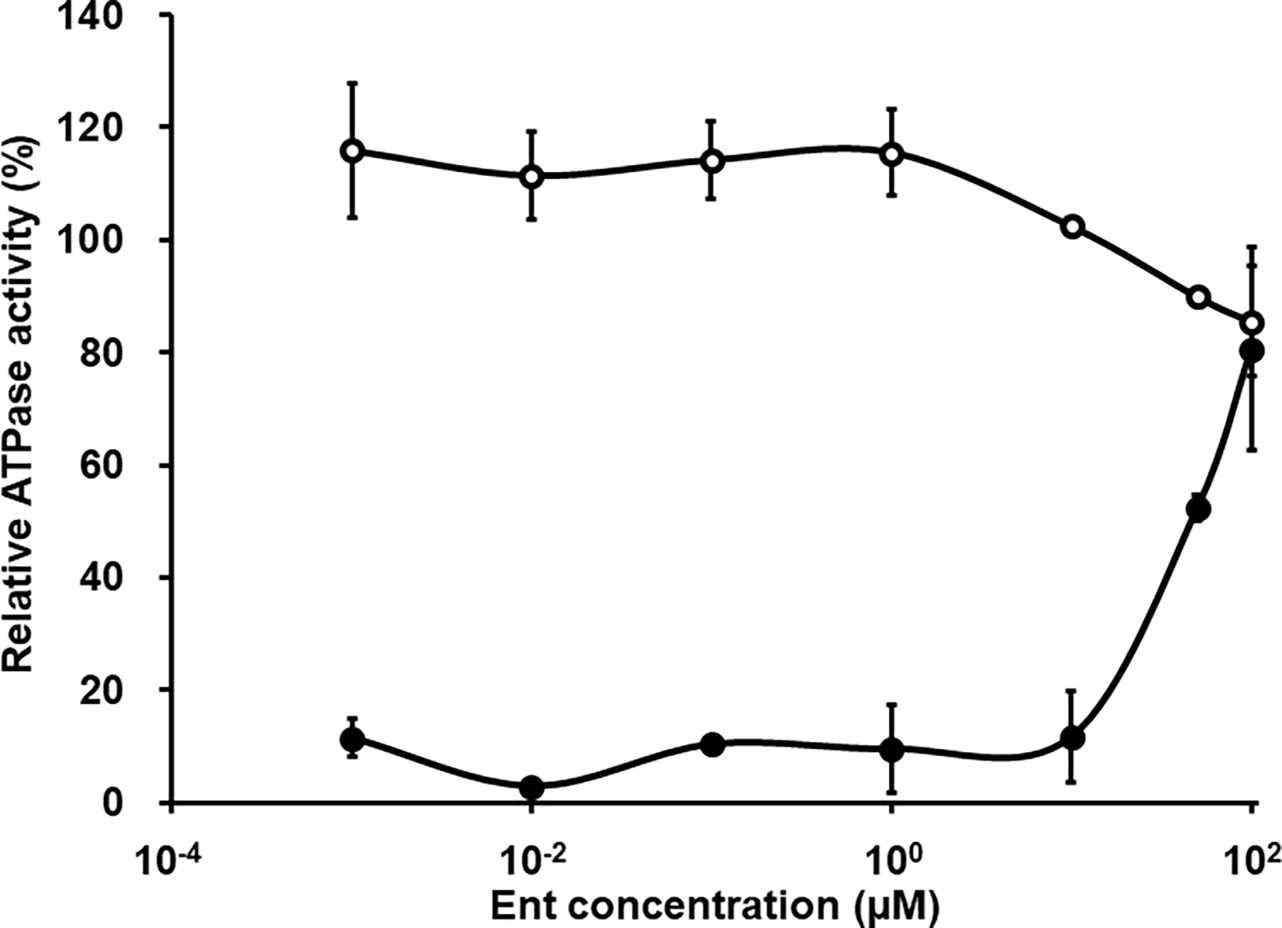
Effects of Ent on ATPase activation and inhibition in P-gp-expressing Sf9 vesicles. P-gp-expressing membrane vesicles were incubated with 1 nM– 100 μM Ent for 10 min at 37°C. Closed circles indicate the results in the activation study and open circles indicate those in the inhibition study. Each point is the mean ± S.D. (n = 2).

Furthermore, we evaluated whether or not Ent is a substrate of P-gp using P-gp-expressing Sf9 vesicles. Activation assay showed that 10 μM or more Ent increased ATPase activity, while in inhibition assay, 10 μM or more Ent inhibited ATPase activity (Fig 6).

## Discussion

In this study, we examined whether or not the EMT inhibitor Ent suppresses P-gp hyper-activation in Snail-overexpressing cells. Various reports have indicated that exposure to high concentrations of cytotoxic agents or HDAC inhibitors induces P-gp mRNA expression [21] [22] [23]. Based on these reports, we explored the concentration range of Ent within which it does not show cytotoxicity or induce AcH3 accumulation, and we found that less than 100 nM Ent had no effect on cytotoxicity or AcH3 accumulation (Fig 1A and 1B). Lillico et al. reported that the IC_50_ values of Ent in K562 (chronic myelogenous leukemia) and HEK293 (human embryonic kidney) cells were 0.97 ± 0.04 μM and 5.4 ± 0.2 μM respectively [24]. Therefore, it is unlikely that Ent would have affected normal cell survival in this study.

Migration assay and mRNA expression analysis both indicated that treatment with 10 nM Ent partially suppressed Snail-induced EMT, as reflected by the reversal of E-cadherin down-regulation and vimentin up-regulation (Fig 2, Fig 3A and 3B), without changing Snail mRNA expression (Fig 3C). Therefore, our data suggest that the reversing effect of Ent on Snail-induced EMT does not depend on suppression of Snail expression.

Under the same conditions, we evaluated P-gp activity using Rho123 efflux and uptake assays. Efflux assays revealed that P-gp efflux activity was significantly increased in Snail-overexpressing cells. Moreover, Ent reduced the Rho123 efflux rate by 36% in Mock cells and by 52% in Snail-overexpressing cells (Fig 4A). In accordance with this, Ent increased Rho123 accumulation in Snail-overexpressing cells to the same level as in Mock cells (Fig 4B). However, P-gp mRNA expression was not changed by Ent in either Mock or Snail-overexpressing cells (Fig 3D). Therefore, the results of Rho123 assays were not due to altered P-gp mRNA expression. These results indicate that Ent preferentially suppresses P-gp activity in Snail-overexpressing cells.

In the present study, we also found that Ent at the concentration of 10 nM is not a substrate of P-gp (Figs 5 and 6). This is important because, although various P-gp inhibitors have been shown to overcome cancer MDR, most of them are substrates of P-gp, and therefore they might cause adverse drug-drug interactions at P-gp in normal organs such as the kidney [25]. Thus, Ent may be a candidate for a non-substrate P-gp inhibitor acting preferentially on cancer cells exhibiting Snail-induced EMT.

In this study, we examined the inhibition of Snail-induced EMT and P-gp activation using 10 nM Ent, which was considered not to have any effect on P-gp mRNA expression. Although Ent sufficiently inhibited P-gp activation, it only partially inhibited EMT in Snail-overexpressing cells. Shah *et al* have reported that 1 μM Ent reversed EMT features in triple-negative breast cancer. Therefore, the use of 10 nM or more Ent should be effective for inhibition of both P-gp activation and EMT. On the other hand, our results also indicated that Ent is a substrate and/or inhibitor of P-gp at concentrations greater than or equal to 10 μM (Fig 6). Further study will be needed to explore the optimal concentration of Ent for inhibition of P-gp activation and EMT only in Snail-overexpressing cells, without inhibiting P-gp function in normal tissues.

Ent treatment inhibited P-gp activation in Snail-overexpressing cells in this study. However, the mechanism involved remains unclear. A previous study revealed that Ent suppresses Snail expression and the binding of Snail to E-cadherin promoter [18]. The binding of Snail to its target gene promoter is required for complex formation with various factors such as HDAC I [26]. In the present study, we used AcH3 accumulation as an indicator of the HDAC inhibition potential of Ent. However, this might not fully reflect the Ent-mediated HDAC inhibition state. Thus, we considered that Ent weakly inhibited HDAC activity, leading to reduced complex formation with Snail and HDAC I. We previously showed that P-gp protein expression of Snail-overexpressing HCC827 is unchanged, even though P-gp activity is up-regulated. These results might indicate that P-gp activation is induced through indirect mechanisms, such as P-gp posttranslational modification. Therefore, Ent might suppress P-gp activation through the modulation of these mechanisms in Snail-overexpressing cells.

It should also be noted that many epidermal growth factor receptor tyrosine kinase inhibitors (EGFR-TKIs; e.g. gefitinib, erlotinib), which are key molecules in non-small cell lung cancer treatment [27] [28] [29] [30], are substrates of P-gp. This may be the reason why treatment of P-gp-mediated MDR cancer with these molecules is often unsuccessful. On the other hand, it was reported that Ent overcomes gefitinib resistance in *vitro* [31]. Moreover, a clinical trial indicated that combined treatment with erlotinib and Ent improved overall survival in non-small cell lung cancer patients who showed high E-cadherin expression levels [15]. Our findings suggest that P-gp inactivation by Ent may underlie this phenomenon, at least in part. Further studies are needed to explore optimal Ent treatment for controlling both EMT and P-gp activity, and to clarify the mechanism of P-gp regulation by Ent.

## References

[1] Jemal A, Center MM, DeSantis C, Ward EM. Global patterns of cancer incidence and mortality rates and trends. Cancer Epidemiol Biomarkers Prev. 2010;19(8):1893–907. Epub 2010/07/22. doi: 10.1158/1055-9965.EPI-10-0437 . 20647400 10.1158/1055-9965.EPI-10-0437

[2] Schouten LJ, Rutten J, Huveneers HA, Twijnstra A. Incidence of brain metastases in a cohort of patients with carcinoma of the breast, colon, kidney, and lung and melanoma. Cancer. 2002;94(10):2698–705. Epub 2002/08/14. . 12173339 10.1002/cncr.10541

[3] Bill R, Christofori G. The relevance of EMT in breast cancer metastasis: Correlation or causality? FEBS Lett. 2015;589(14):1577–87. Epub 2015/05/17. doi: 10.1016/j.febslet.2015.05.002 . 25979173 10.1016/j.febslet.2015.05.002

[4] Labelle M, Begum S, Hynes RO. Direct signaling between platelets and cancer cells induces an epithelial-mesenchymal-like transition and promotes metastasis. Cancer Cell. 2011;20(5):576–90. Epub 2011/11/19. doi: 10.1016/j.ccr.2011.09.009 ; PubMed Central PMCID: PMCPmc3487108. 22094253 10.1016/j.ccr.2011.09.009 PMC3487108

[5] Lorenz KJ, Kraft K, Graf F, Propper C, Steinestel K. Role of reflux-induced epithelial-mesenchymal transition in periprosthetic leakage after prosthetic voice rehabilitation. Head Neck. 2015;37(4):530–6. Epub 2014/02/18. doi: 10.1002/hed.23622 . 24532155 10.1002/hed.23622

[6] Zhu Y, Nilsson M, Sundfeldt K. Phenotypic plasticity of the ovarian surface epithelium: TGF-beta 1 induction of epithelial to mesenchymal transition (EMT) in vitro. Endocrinology. 2010;151(11):5497–505. Epub 2010/09/17. doi: 10.1210/en.2010-0486 . 20844000 10.1210/en.2010-0486

[7] Lioni M, Brafford P, Andl C, Rustgi A, El-Deiry W, Herlyn M, et al Dysregulation of claudin-7 leads to loss of E-cadherin expression and the increased invasion of esophageal squamous cell carcinoma cells. Am J Pathol. 2007;170(2):709–21. Epub 2007/01/27. doi: 10.2353/ajpath.2007.060343 ; PubMed Central PMCID: PMCPmc1851859. 17255337 10.2353/ajpath.2007.060343 PMC1851859

[8] Kagalwalla AF, Akhtar N, Woodruff SA, Rea BA, Masterson JC, Mukkada V, et al Eosinophilic esophagitis: epithelial mesenchymal transition contributes to esophageal remodeling and reverses with treatment. J Allergy Clin Immunol. 2012;129(5):1387–96.e7. Epub 2012/04/03. doi: 10.1016/j.jaci.2012.03.005 ; PubMed Central PMCID: PMCPmc3340537. 22465212 10.1016/j.jaci.2012.03.005 PMC3340537

[9] Sanchez-Tillo E, de Barrios O, Siles L, Amendola PG, Darling DS, Cuatrecasas M, et al ZEB1 Promotes invasiveness of colorectal carcinoma cells through the opposing regulation of uPA and PAI-1. Clin Cancer Res. 2013;19(5):1071–82. Epub 2013/01/24. doi: 10.1158/1078-0432.CCR-12-2675 . 23340304 10.1158/1078-0432.CCR-12-2675

[10] Barrallo-Gimeno A, Nieto MA. The Snail genes as inducers of cell movement and survival: implications in development and cancer. Development (Cambridge, England). 2005;132(14):3151–61. Epub 2005/06/29. doi: 10.1242/dev.01907 . 15983400 10.1242/dev.01907

[11] Fischer KR, Durrans A, Lee S, Sheng J, Li F, Wong ST, et al Epithelial-to-mesenchymal transition is not required for lung metastasis but contributes to chemoresistance. Nature. 2015;527(7579):472–6. Epub 2015/11/13. doi: 10.1038/nature15748 ; PubMed Central PMCID: PMCPMC4662610. 26560033 10.1038/nature15748 PMC4662610

[12] Tomono T, Yano K, Ogihara T. Snail-Induced Epithelial-to-Mesenchymal Transition Enhances P-gp-Mediated Multidrug Resistance in HCC827 Cells. Journal of pharmaceutical sciences. 2017 Epub 2017/03/23. doi: 10.1016/j.xphs.2017.03.011 . 28322937 10.1016/j.xphs.2017.03.011

[13] Amin ML. P-glycoprotein Inhibition for Optimal Drug Delivery. Drug Target Insights. 2013;7:27–34. Epub 2013/09/12. doi: 10.4137/DTI.S12519 ; PubMed Central PMCID: PMCPmc3762612. 24023511 10.4137/DTI.S12519 PMC3762612

[14] Yardley DA, Ismail-Khan RR, Melichar B, Lichinitser M, Munster PN, Klein PM, et al Randomized phase II, double-blind, placebo-controlled study of exemestane with or without entinostat in postmenopausal women with locally recurrent or metastatic estrogen receptor-positive breast cancer progressing on treatment with a nonsteroidal aromatase inhibitor. J Clin Oncol. 2013;31(17):2128–35. Epub 2013/05/08. doi: 10.1200/JCO.2012.43.7251 ; PubMed Central PMCID: PMCPmc4881332. 23650416 10.1200/JCO.2012.43.7251 PMC4881332

[15] Witta SE, Jotte RM, Konduri K, Neubauer MA, Spira AI, Ruxer RL, et al Randomized phase II trial of erlotinib with and without entinostat in patients with advanced non-small-cell lung cancer who progressed on prior chemotherapy. J Clin Oncol. 2012;30(18):2248–55. Epub 2012/04/18. doi: 10.1200/JCO.2011.38.9411 ; PubMed Central PMCID: PMCPmc4798782. 22508830 10.1200/JCO.2011.38.9411 PMC4798782

[16] Hauschild A, Trefzer U, Garbe C, Kaehler KC, Ugurel S, Kiecker F, et al Multicenter phase II trial of the histone deacetylase inhibitor pyridylmethyl-N-{4-[(2-aminophenyl)-carbamoyl]-benzyl}-carbamate in pretreated metastatic melanoma. Melanoma Res. 2008;18(4):274–8. Epub 2008/07/16. doi: 10.1097/CMR.0b013e328307c248 . 18626312 10.1097/CMR.0b013e328307c248

[17] Ngamphaiboon N, Dy GK, Ma WW, Zhao Y, Reungwetwattana T, DePaolo D, et al A phase I study of the histone deacetylase (HDAC) inhibitor entinostat, in combination with sorafenib in patients with advanced solid tumors. Invest New Drugs. 2015;33(1):225–32. Epub 2014/11/06. doi: 10.1007/s10637-014-0174-6 . 25371323 10.1007/s10637-014-0174-6

[18] Shah P, Gau Y, Sabnis G. Histone deacetylase inhibitor entinostat reverses epithelial to mesenchymal transition of breast cancer cells by reversing the repression of E-cadherin. Breast cancer research and treatment. 2014;143(1):99–111. Epub 2013/12/07. doi: 10.1007/s10549-013-2784-7 . 24305977 10.1007/s10549-013-2784-7

[19] Kajita M, McClinic KN, Wade PA. Aberrant expression of the transcription factors snail and slug alters the response to genotoxic stress. Mol Cell Biol. 2004;24(17):7559–66. Epub 2004/08/18. doi: 10.1128/MCB.24.17.7559-7566.2004 ; PubMed Central PMCID: PMCPmc506998. 15314165 10.1128/MCB.24.17.7559-7566.2004 PMC506998

[20] Gonzalez ML, Vera DMA, Laiolo J, Joray MB, Maccioni M, Palacios SM, et al Mechanism Underlying the Reversal of Drug Resistance in P-Glycoprotein-Expressing Leukemia Cells by Pinoresinol and the Study of a Derivative. Frontiers in pharmacology. 2017;8:205 Epub 2017/05/11. doi: 10.3389/fphar.2017.00205 ; PubMed Central PMCID: PMCPMC5403950. 28487651 10.3389/fphar.2017.00205 PMC5403950

[21] Tsou SH, Chen TM, Hsiao HT, Chen YH. A critical dose of doxorubicin is required to alter the gene expression profiles in MCF-7 cells acquiring multidrug resistance. PloS one. 2015;10(1):e0116747 Epub 2015/01/31. doi: 10.1371/journal.pone.0116747 ; PubMed Central PMCID: PMCPMC4312059. 25635866 10.1371/journal.pone.0116747 PMC4312059

[22] Tabe Y, Konopleva M, Contractor R, Munsell M, Schober WD, Jin L, et al Up-regulation of MDR1 and induction of doxorubicin resistance by histone deacetylase inhibitor depsipeptide (FK228) and ATRA in acute promyelocytic leukemia cells. Blood. 2006;107(4):1546–54. Epub 2005/10/15. doi: 10.1182/blood-2004-10-4126 ; PubMed Central PMCID: PMCPMC1895410. 16223781 10.1182/blood-2004-10-4126 PMC1895410

[23] Xu Y, Jiang Z, Yin P, Li Q, Liu J. Role for Class I histone deacetylases in multidrug resistance. Experimental cell research. 2012;318(3):177–86. Epub 2011/12/14. doi: 10.1016/j.yexcr.2011.11.010 . 22154511 10.1016/j.yexcr.2011.11.010

[24] Lillico R, Sobral MG, Stesco N, Lakowski TM. HDAC inhibitors induce global changes in histone lysine and arginine methylation and alter expression of lysine demethylases. Journal of proteomics. 2016;133:125–33. Epub 2016/01/02. doi: 10.1016/j.jprot.2015.12.018 . 26721445 10.1016/j.jprot.2015.12.018

[25] Sharom FJ. The P-glycoprotein multidrug transporter. Essays Biochem. 2011;50(1):161–78. Epub 2011/10/05. doi: 10.1042/bse0500161 . 21967057 10.1042/bse0500161

[26] Peinado H, Ballestar E, Esteller M, Cano A. Snail mediates E-cadherin repression by the recruitment of the Sin3A/histone deacetylase 1 (HDAC1)/HDAC2 complex. Molecular and cellular biology. 2004;24(1):306–19. Epub 2003/12/16. doi: 10.1128/MCB.24.1.306-319.2004 ; PubMed Central PMCID: PMCPMC303344. 14673164 10.1128/MCB.24.1.306-319.2004 PMC303344

[27] Hynes NE, Lane HA. ERBB receptors and cancer: the complexity of targeted inhibitors. Nat Rev Cancer. 2005;5(5):341–54. Epub 2005/05/03. doi: 10.1038/nrc1609 . 15864276 10.1038/nrc1609

[28] Ciuleanu T, Stelmakh L, Cicenas S, Miliauskas S, Grigorescu AC, Hillenbach C, et al Efficacy and safety of erlotinib versus chemotherapy in second-line treatment of patients with advanced, non-small-cell lung cancer with poor prognosis (TITAN): a randomised multicentre, open-label, phase 3 study. Lancet Oncol. 2012;13(3):300–8. Epub 2012/01/27. doi: 10.1016/S1470-2045(11)70385-0 . 22277837 10.1016/S1470-2045(11)70385-0

[29] Collins DM, Crown J, O'Donovan N, Devery A, O'Sullivan F, O'Driscoll L, et al Tyrosine kinase inhibitors potentiate the cytotoxicity of MDR-substrate anticancer agents independent of growth factor receptor status in lung cancer cell lines. Invest New Drugs. 2010;28(4):433–44. Epub 2009/06/06. doi: 10.1007/s10637-009-9266-0 . 19499189 10.1007/s10637-009-9266-0

[30] Marchetti S, de Vries NA, Buckle T, Bolijn MJ, van Eijndhoven MA, Beijnen JH, et al Effect of the ATP-binding cassette drug transporters ABCB1, ABCG2, and ABCC2 on erlotinib hydrochloride (Tarceva) disposition in in vitro and in vivo pharmacokinetic studies employing Bcrp1-/-/Mdr1a/1b-/- (triple-knockout) and wild-type mice. Mol Cancer Ther. 2008;7(8):2280–7. Epub 2008/08/30. doi: 10.1158/1535-7163.MCT-07-2250 . 18723475 10.1158/1535-7163.MCT-07-2250

[31] Witta SE, Gemmill RM, Hirsch FR, Coldren CD, Hedman K, Ravdel L, et al Restoring E-cadherin expression increases sensitivity to epidermal growth factor receptor inhibitors in lung cancer cell lines. Cancer Res. 2006;66(2):944–50. Epub 2006/01/21. doi: 10.1158/0008-5472.CAN-05-1988 . 16424029 10.1158/0008-5472.CAN-05-1988

